# Paradoxical Sinus Bradycardia in the Setting of Acute Cardiac Tamponade: A Case Report

**DOI:** 10.7759/cureus.105825

**Published:** 2026-03-25

**Authors:** Cameron Bondy, Sondos Badran, Lucia Schroeder, Nicolas Thor

**Affiliations:** 1 Internal Medicine, Arrowhead Regional Medical Center, Colton, USA; 2 Cardiology, Arrowhead Regional Medical Center, Colton, USA

**Keywords:** acute pericarditis, cardiac tamponade, case report, paradoxical sinus bradycardia, pericardial effusion, pericardiocentesis

## Abstract

Cardiac tamponade is a life-threatening condition classically associated with compensatory sinus tachycardia due to impaired diastolic filling and reduced cardiac output; however, paradoxical sinus bradycardia is rare and may delay recognition. We report a case of a 43-year-old woman with obesity status post gastric sleeve, asthma, and hyperlipidemia who presented with two weeks of progressive dyspnea, pleuritic chest pain, and chest tightness following a suspected viral syndrome, with symptoms worsening supine and improving when leaning forward. Initial evaluation demonstrated muffled heart sounds, sinus rhythm with electrical alternans on electrocardiography, cardiomegaly on chest radiography, and a large pericardial effusion on point-of-care ultrasound without initial tamponade physiology. While awaiting further workup, she developed recurrent syncope with profound sinus bradycardia and transient pulselessness requiring vasopressor support. Transthoracic echocardiography revealed right atrial compression and right ventricular diastolic collapse consistent with acute cardiac tamponade. Urgent pericardiocentesis drained 1.3 liters of bloody, turbid fluid with immediate resolution of bradycardia and hemodynamic instability. Pericardial fluid analysis was exudative with inflammatory cells, and there was no evidence of malignancy. The patient was successfully treated with high-dose aspirin and colchicine without recurrence. Notably, the absence of compensatory tachycardia in this case contributed to delayed clinical recognition of tamponade physiology. This case highlights that cardiac tamponade may rarely present with paradoxical sinus bradycardia rather than tachycardia, underscoring the importance of maintaining clinical suspicion and performing prompt echocardiographic assessment, as urgent pericardial drainage can be life-saving even in nonclassical presentations.

## Introduction

Cardiac tamponade classically presents with sinus tachycardia as a compensatory response to reduced cardiac output resulting from impaired ventricular diastolic filling and decreased stroke volume. As intrapericardial pressure rises, preload falls, and tachycardia serves as a critical mechanism to maintain cardiac output in the setting of hemodynamic compromise [1,2]. In contrast, the presence of sinus bradycardia represents a paradoxical and atypical presentation. Bradycardia is physiologically discordant with the expected compensatory response and may obscure clinical recognition of tamponade, particularly when other classic findings such as hypotension or elevated jugular venous pressure are subtle or incomplete. Importantly, the absence of compensatory tachycardia may reduce clinical suspicion for tamponade and contribute to delays in diagnosis and definitive management. Prior reports have described paradoxical sinus bradycardia in acute cardiac tamponade, hypothesized to result from heightened vagal tone, reflex autonomic responses to ventricular underfilling, or ischemia of the conduction system [3]. This abnormal chronotropic response may misdirect clinicians toward alternative diagnoses and delay definitive imaging and life-saving intervention with pericardial drainage, thereby increasing morbidity and mortality [2,3].

## Case presentation

A 43-year-old woman with a history of obesity status post gastric sleeve, asthma, and hyperlipidemia presented with two weeks of progressive dyspnea, chest tightness, and pleuritic chest pain following a suspected viral syndrome. She described the onset of symptoms approximately one month prior to her presentation in the emergency department. Symptoms were exacerbated in the supine position and improved with forward leaning. Her home medications include pantoprazole and simvastatin. 

**Figure 1:**
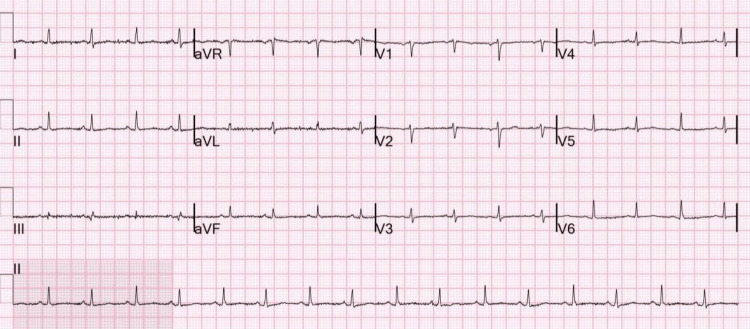
Initial ECG Initial ECG demonstrating sinus rhythm with a relatively slow ventricular rate. P waves preceded each QRS complex with a normal PR interval, narrow QRS duration, and no significant QT prolongation. Diffuse low QRS voltage was present across both limbs and precordial leads. Beat-to-beat variation in QRS amplitude consistent with electrical alternans was noted, most apparent on the rhythm strip and sequential leads. There were no focal ST-segment elevations or depressions and no clear diffuse concave ST elevations or PR depressions. In the appropriate clinical context, these findings were highly suggestive of a large pericardial effusion and impending cardiac tamponade.

**Figure 2:**
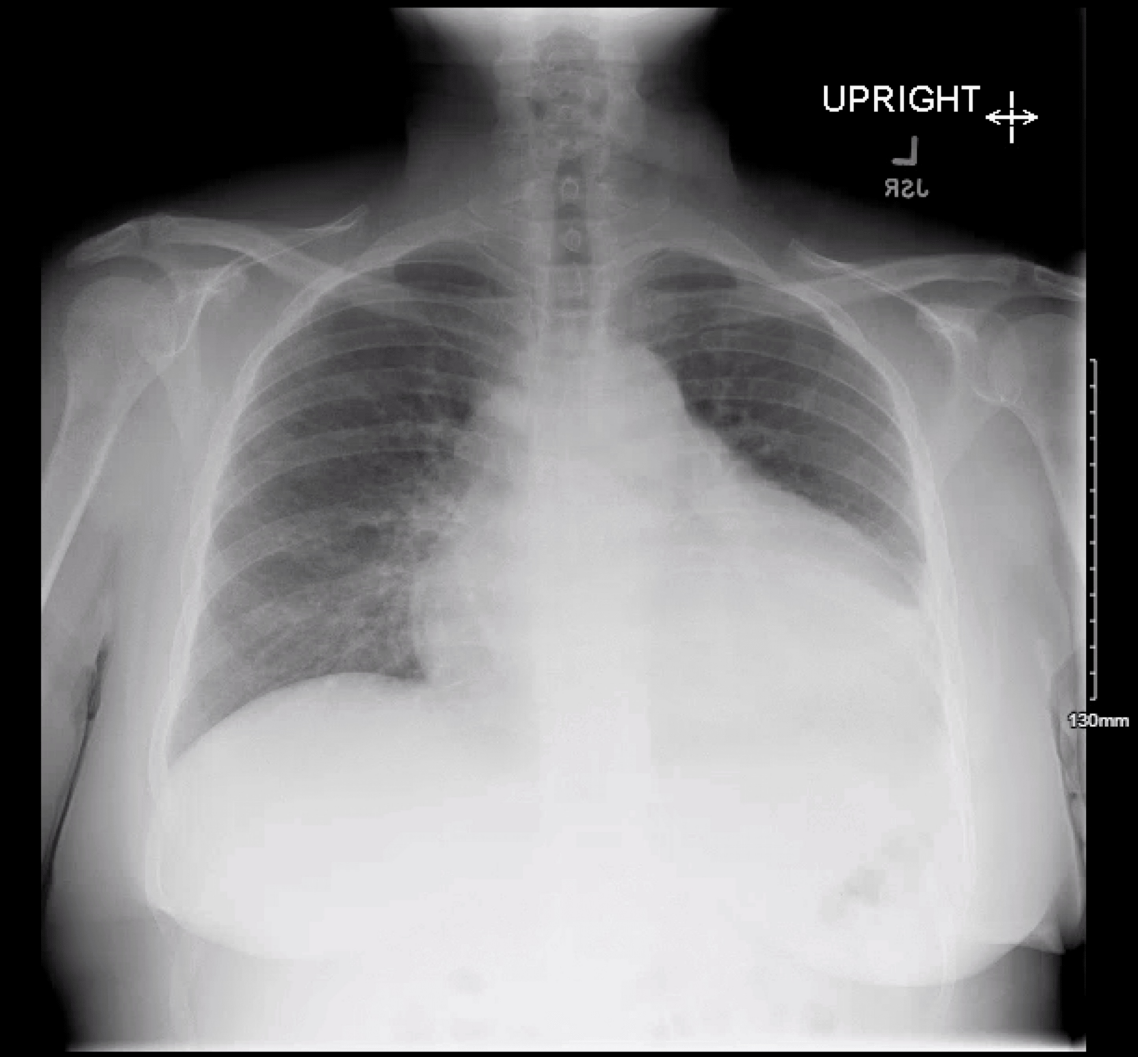
Initial chest X-ray The cardiomediastinal silhouette is markedly enlarged with a smooth, globular configuration, raising concern for a large pericardial effusion.

On examination, the patient had an initial heart rate of 40-50 beats per minute with normotensive blood pressure. Cardiac auscultation revealed muffled heart sounds. Electrocardiography demonstrated sinus rhythm with electrical alternans (Figure 1; ECG 1), and chest radiography (Figure 2) showed cardiomegaly, findings consistent with a large pericardial effusion.

Her initial lab-work evaluation is described in Table 1. Results demonstrated a microcytic, hypochromic anemia with mild anisocytosis and an associated reactive thrombocytosis. The white blood cell count is overall within normal limits with a largely unremarkable differential. The metabolic panel is notable for a mild decrease in bicarbonate and borderline low calcium, with otherwise preserved renal function and unremarkable electrolytes. Liver studies are largely within normal limits, though there is a mild isolated elevation in alkaline phosphatase with normal transaminases and bilirubin. Cardiac biomarkers are negative, and thyroid function is within normal limits. 

**Table 1:** Initial lab results WBC, white blood cells; RBC, red blood cells; MCV, mean corpuscular volume; MCH, mean corpuscular hemoglobin; RDW, red cell distribution width; MPV, mean platelet volume; MCHC, mean corpuscular hemoglobin concentration; nRBC, nucleated RBC; BUN, blood urea nitrogen; eGFR, estimated glomerular filtration rate; AST, aspartate aminotransferase; ALT, alanine aminotransferase; TSH, thyroid-stimulating hormone.

Test	Value	Reference Range
WBC (×10^3^/µL)	10.1	4.0-11.0
RBC (×10^6^/µL)	4.13	4.2-5.9 (M), 3.8-5.1 (F)
Hemoglobin (g/dL)	9.4	13.5-17.5 (M), 12.0-15.5 (F)
Hematocrit (%)	31	41-53 (M), 36-46 (F)
MCV (fL)	75	80-100
MCH (pg)	22.8	27-33
RDW (%)	15	11.5-14.5
Platelets (×10^3^/µL)	409	150-400
MPV (fL)	9.2	7.5-11.5
MCHC (g/dL)	30	32-36
Neutrophils (%)	67	40-70
Lymphocytes (%)	23	20-40
Monocytes (%)	8	2-8
Eosinophils (%)	1	1-4
Basophils (%)	1	0-1
Neutrophils absolute (×10^3^/µL)	6.76	1.5-7.5
Lymphocytes absolute (×10^3^/µL)	2.34	1.0-4.0
Monocytes absolute (×10^3^/µL)	0.84	0.2-0.8
Eosinophils absolute (×10^3^/µL)	0.06	0.0-0.5
Basophils absolute (×10^3^/µL)	0.05	0.0-0.2
nRBC	0	0
Immature granulocytes (%)	0.4	0-0.4
Immature granulocytes absolute (×10^3^/µL)	0.04	0.0-0.03
Sodium (mmol/L)	139	135-145
Potassium (mmol/L)	3.9	3.5-5.0
Chloride (mmol/L)	105	98-106
CO_2_ (mmol/L)	21	22-29
BUN (mg/dL)	14	7-20
Creatinine (mg/dL)	0.71	0.6-1.3
eGFR (mL/min/1.73 m²)	108.3	>60
Glucose (mg/dL)	110	70-99 (fasting)
Calcium (mg/dL)	8.1	8.5-10.5
Phosphorus (mg/dL)	2.9	2.5-4.5
Magnesium (mg/dL)	2.1	1.7-2.2
Alkaline phosphatase (U/L)	134	44-147
Albumin (g/dL)	3.6	3.5-5.0
Total protein (g/dL)	6.4	6.0-8.3
AST (U/L)	22	10-40
ALT (U/L)	33	7-56
Direct bilirubin (mg/dL)	<0.2	0.0-0.3
Total bilirubin (mg/dL)	0.4	0.1-1.2
Troponin T (ng/L)	<6.00	<14 (high sensitivity)
TSH (µIU/mL)	1.18	0.4-4.0

Point-of-care ultrasound identified a large circumferential pericardial effusion without overt tamponade physiology.

Laboratory evaluation was notable for a hemoglobin level of 9.4 g/dL and a positive antinuclear antibody (ANA) titer of 1:320. While awaiting further diagnostic evaluation, the patient developed recurrent syncopal episodes associated with profound sinus bradycardia and transient pulselessness, requiring vasopressor support.

**Figure 3:**
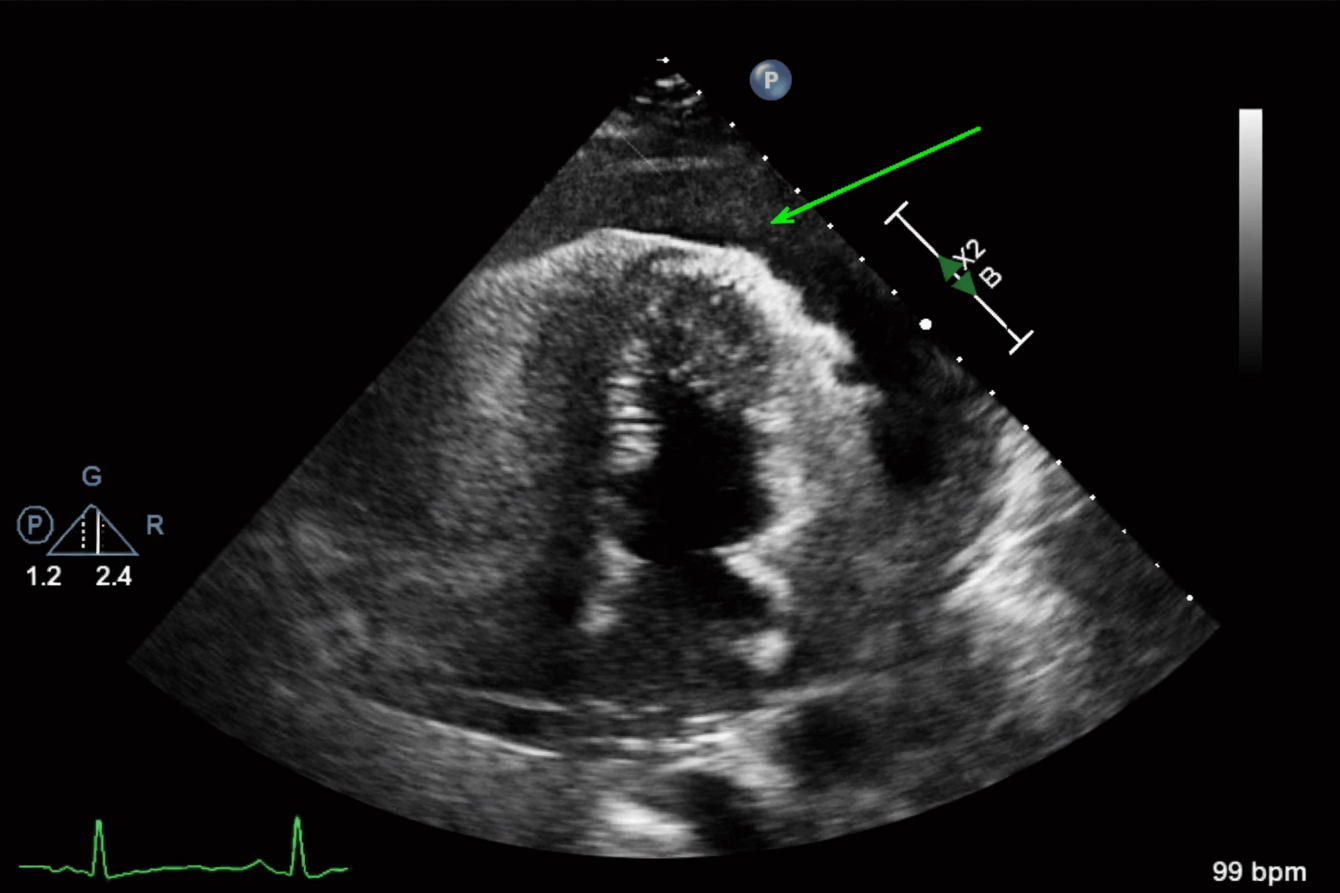
Initial transthoracic echocardiogram (apical four-chamber view) The initial transthoracic echocardiogram demonstrated a large circumferential pericardial effusion, most prominent along the anterior and lateral aspects of the heart. The green arrow highlights a substantial anechoic fluid collection separating the visceral and parietal pericardium, consistent with a large pericardial effusion. The left ventricle appeared hyperdynamic with preserved systolic function, while there was evidence of right-sided chamber compression, particularly involving the right atrium and early diastolic right ventricular collapse, findings concerning for evolving cardiac tamponade physiology. These echocardiographic features, in conjunction with the patient’s clinical deterioration, supported the diagnosis of acute cardiac tamponade and prompted urgent pericardial drainage.

Formal transthoracic echocardiography (TTE) demonstrated a hyperdynamic left ventricle with right atrial compression and right ventricular diastolic collapse, findings consistent with acute cardiac tamponade (Figure 3; TTE 1).

**Figure 4:**
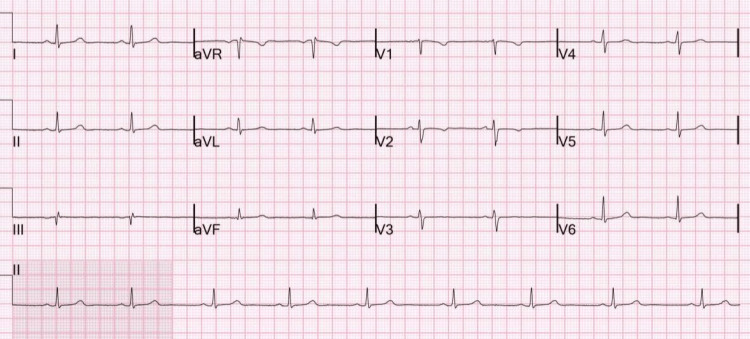
Repeat ECG showing interval improvement compared to the prior study with resolution of previously noted electrical alternans

**Figure 5:**
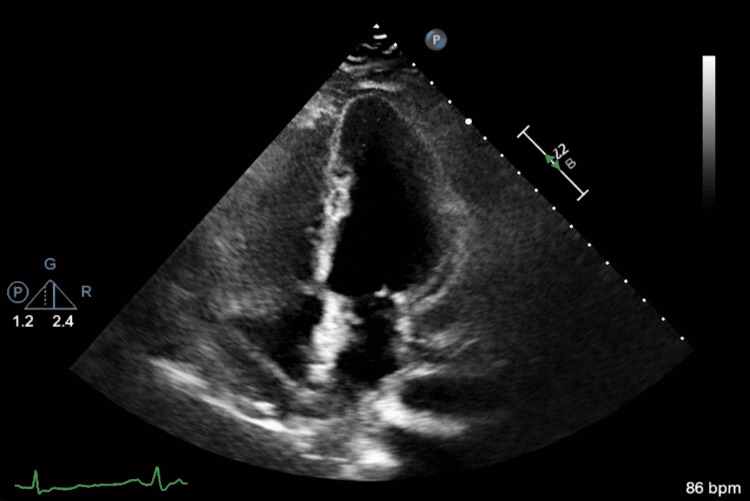
Repeat transthoracic echocardiogram (apical four-chamber view) The follow-up transthoracic echocardiogram obtained after pericardiocentesis demonstrated near-complete resolution of the previously large pericardial effusion, with no significant residual circumferential fluid visualized. The cardiac chambers were no longer compressed, and there was resolution of right atrial and right ventricular diastolic collapse, indicating normalization of intracardiac filling. Left ventricular systolic function remained preserved with appropriate chamber dimensions and no evidence of tamponade physiology. Overall, this study confirmed effective pericardial drainage with restoration of normal cardiac mechanics, correlating with the patient’s clinical and hemodynamic improvement.

Urgent pericardiocentesis was performed, with drainage of 1.3 liters of bloody, turbid fluid, followed by immediate resolution of bradycardia and hemodynamic instability. Pericardial fluid analysis revealed an exudative effusion with reactive mesothelial cells and inflammatory cells on cytology, without evidence of malignancy. The patient was treated with high-dose aspirin and colchicine. Repeat electrocardiography and TTE demonstrated interval improvement (Figures 4, 5). The patient was subsequently discharged with near-resolution of symptoms.

Despite extensive diagnostic evaluation, no definitive etiology for the pericardial effusion was identified. Infectious and autoimmune testing was unrevealing, including a negative respiratory pathogen panel, negative viral hepatitis serologies, negative HIV and syphilis testing, and a negative rheumatoid factor. No evidence of malignancy was identified on pericardial fluid analysis, which is described in Table 2. 

**Table 2:** Pericardial fluid analysis LDH, lactate dehydrogenase; N/A, not applicable.

Parameter	Value	Reference/Expected
Color	Red	Clear to straw-colored
Appearance	Turbid	Clear
Neutrophils (%)	57	<25
Lymphocytes (%)	15	Variable (often low unless chronic)
Monocytes/Macrophages (%)	28	Present in small amounts
Glucose (fluid) (mg/dL)	80	~Serum glucose (typically >60)
LDH (fluid) (U/L)	378	Low; typically <200 or <2/3 serum LDH
pH (fluid)	8	7.60-7.64 (slightly alkaline)
Protein (fluid) (g/dL)	4.5	Low (<3.0 in transudate)
Albumin (fluid)	4.3	Low; compared with serum for gradient
Fluid type	Pericardial fluid	N/A
Total cells counted	100	N/A
Cytology results	Reactive mesothelial cells, neutrophils, and lymphocytes. No malignant cells identified.	

## Discussion

Cardiac tamponade is traditionally associated with sinus tachycardia as a compensatory response to reduced cardiac output resulting from impaired ventricular filling and decreased stroke volume [1,2]. In this context, sinus bradycardia is rare and counterintuitive, with only a limited number of cases described in the literature [3]. The presence of bradycardia may therefore obscure recognition of tamponade and delay definitive intervention.

Several mechanisms have been proposed to explain paradoxical bradycardia in cardiac tamponade. One hypothesis involves increased vagal tone secondary to pericardial stretch or irritation, resulting in suppression of sinoatrial node automaticity. Abrupt pericardial distension may activate cardiac mechanoreceptors and trigger parasympathetic reflexes analogous to the Bezold-Jarisch reflex, leading to inappropriate bradycardia in the setting of acute hemodynamic stress. Additionally, severe right ventricular compression with marked preload reduction may paradoxically augment vagal output rather than stimulate the expected sympathetic response [2,3].

Another proposed mechanism involves transient autonomic imbalance during acute hemodynamic compromise, potentially related to altered baroreceptor signaling or transient hypoperfusion of the cardiac conduction system [1]. The rapid and complete resolution of sinus bradycardia following pericardiocentesis in this case supports a reflex-mediated and reversible process rather than intrinsic conduction system disease, as has been described in prior reports [3].

This case underscores the importance of maintaining a high index of suspicion for cardiac tamponade even in the absence of classic features such as tachycardia or hypotension. Electrocardiographic findings of electrical alternans, radiographic cardiomegaly, positional chest pain, and echocardiographic evidence of chamber compression remain key diagnostic clues [1,4,5]. Point-of-care ultrasound is particularly valuable for the rapid identification of large pericardial effusions and evolving tamponade physiology, facilitating early diagnosis and timely intervention [4,5].

The bloody, exudative nature of the pericardial effusion, elevated ANA titer, and antecedent viral syndrome are consistent with an inflammatory etiology, although malignant, infectious, and autoimmune causes must be systematically excluded. Current guidelines recommend anti-inflammatory therapy with nonsteroidal anti-inflammatory agents and colchicine following pericardial drainage in cases of presumed inflammatory pericarditis to reduce recurrence risk, as was implemented in this patient [4,6].

## Conclusions

This case illustrates that acute cardiac tamponade may rarely present with paradoxical sinus bradycardia and syncope rather than the expected compensatory tachycardia. Although sinus tachycardia is considered a hallmark physiologic response to reduced preload and impaired cardiac output in tamponade, prior reports have demonstrated that bradyarrhythmias can occur in select cases and may represent an atypical autonomic or reflex-mediated response to acute pericardial pressure elevation. Such nonclassical presentations may obscure clinical recognition, particularly when hypotension is absent or when compensatory mechanisms are blunted, thereby increasing the risk of diagnostic delay and sudden hemodynamic deterioration.

Importantly, the absence of tachycardia does not exclude the diagnosis of cardiac tamponade. Clinicians should maintain a high index of suspicion in patients with compatible symptoms, electrocardiographic findings, or imaging evidence of pericardial effusion, even when vital signs appear discordant with classic teaching. Echocardiography remains the diagnostic modality of choice, as it provides rapid identification of chamber compression and diastolic collapse, which are central to the diagnosis regardless of heart rate response.

Prompt pericardial drainage remains the definitive treatment for hemodynamically significant tamponade and can result in immediate reversal of both bradyarrhythmia and circulatory compromise. Prior studies and case reports have demonstrated rapid normalization of heart rate and blood pressure following pericardiocentesis, underscoring the mechanical rather than primary electrical nature of the arrhythmia in these cases. Recognition of paradoxical sinus bradycardia as a rare but clinically important manifestation of cardiac tamponade is therefore essential to avoid delayed intervention and prevent potentially catastrophic outcomes.

## References

[1] Spodick DH (2003). Acute cardiac tamponade. N Engl J Med.

[2] Reddy PS, Curtiss EI, O'Toole JD, Shaver JA (1978). Cardiac tamponade: Hemodynamic observations in man. Circulation.

[3] Kostreva DR, Castaner A, Pedersen DH, Kampine JP (1981). Nonvagally mediated bradycardia during cardiac tamponade or severe hemorrhage. Cardiology.

[4] Adler Y, Charron P, Imazio M (2015). 2015 ESC Guidelines for the diagnosis and management of pericardial diseases: The Task Force for the Diagnosis and Management of Pericardial Diseases of the European Society of Cardiology (ESC) Endorsed by: The European Association for Cardio-Thoracic Surgery (EACTS). Eur Heart J.

[5] Tsang TS, Oh JK, Seward JB (1999). Diagnosis and management of cardiac tamponade in the era of echocardiography. Clin Cardiol.

[6] Jensen JK, Poulsen SH, Mølgaard H (2017). Cardiac tamponade: A clinical challenge. Eur Heart J Cardiovasc Imaging.

